# New challenges and opportunities for industrial biotechnology

**DOI:** 10.1186/1475-2859-11-111

**Published:** 2012-08-20

**Authors:** Guo-Qiang Chen

**Affiliations:** 1 MOE Key Lab of Bioinformatics and Systems Biology, Department of Biological Science and Biotechnology, School of Life Sciences, Tsinghua-Peking Center for Life Sciences, Tsinghua University, Beijing, 100084, China; 2 Center for Nano and Micro Mechanics, Tsinghua University, Beijing, 100084, China

**Keywords:** Industrial biotechnology, Shale gas, Oil fields, PHB, Bioplastics, Biofuels, Bulk chemicals

## Abstract

Industrial biotechnology has not developed as fast as expected due to some challenges including the emergences of alternative energy sources, especially shale gas, natural gas hydrate (or gas hydrate) and sand oil et al. The weaknesses of microbial or enzymatic processes compared with the chemical processing also make industrial biotech products less competitive with the chemical ones. However, many opportunities are still there if industrial biotech processes can be as similar as the chemical ones. Taking advantages of the molecular biology and synthetic biology methods as well as changing process patterns, we can develop bioprocesses as competitive as chemical ones, these including the minimized cells, open and continuous fermentation processes et al.

## 

The commercialization of industrial biotechnology is not as fast as we expected. Originally, we believe that production of bulk chemicals including biofuels, polymeric materials and chemical agents using microorganisms or enzymes will provide low cost, environmentally friendly products to partially replace petro-chemicals products [1]. However, this looks not so easy to materialize due to the facts that:

1. Petroleum does not rise in price too much after 2008 financial crisis, other alternative energy sources, especially shale gas, natural gas hydrate (or gas hydrate) and sand oil, have been discovered in large amount and their exploitations are increasingly moving toward a very competitive price;

2. The exhaustion of petroleum seems to be a remote reality

3. Agriculture raw materials for bioprocessing are becoming increasingly costly

4. Low cost raw material cellulose can not be easily used for microbial processes at least for the next 5–10 years

5. Bioprocessing is still not as effective as chemical processing, resulting in high cost of bio-products (Table 1)

6. Bioprocessing that requires large amount of fresh water has had increasing concerns in many water shortage areas

7. The chemical industry is also evolving competitive in various ways including environmentally friendliness, the use of renewable resources (biomass) for making chemicals that are normally derived from petro-chemicals

8. The rapid development of C1 chemical engineering products

9. Large amount of funding is not more directed to industrial biotechnology.

**Table 1:** Comparison of industrial biotechnology and chemical technology

**Items**	**Industrial biotechnology**	**Chemical technology**
Reaction Time	Slow: production takes days	Fast: production takes hours
Substrates	Agricultural products	Petroleum or its derivatives
Conversion of substrates to products	Low: e.g. PHB/glucose ≈ 33 wt% PHA/fatty acids ≈ 60 wt%	High: e.g. Polyethylene/ethylene ≈ 100%
Medium	Water	Mostly organic solvents
Consumption of water	A lot	Less
Reaction conditions	30-40°C, normal pressure	Generally >100°C, High Pressures
Product concentration	Low: Several mg to 100 g/L	Very high
Product recovery cost	Very high	Low to medium
Processing	Normally discontinuous one	Can be continuous
Sterilization	Necessary	No need
Production facility cost	Very high	Low to high (explosive proof)
Waste water	Not toxic, easier to treat	Generally toxic, difficult to treat

Taking the example of polyhydroxyalkanoates (PHA), a biopolyester family that has been exploited to become an industrial value chain [2-4], PHA has not been able to commercially produce in large scale due to the difficulty to lower the production cost especially for their applications as bioplastics that are considered as biodegradable and bio-based despite the possibility of using CO_2_ as substrate [5].

To successfully commercialize PHA, we must keep working hard on the “high volume and low price” strategy by developing better PHA production strains and cost competitive processes. While for some special applications, “low volumes and high price” can be applied, such as products to be used for biomedical purposes, specialty polymers [6,7], chiral monomers, drug development and special applications et al. [8,9]. And this is generally true in order to survive this competitive environment for industrial biotechnology, it must be competitive with the chemical industry. Let’s see what we can do to make this happen. In addition, it is also important to be able to develop processes that combined the advantages of chemical industry to supplement the weaknesses of industrial biotechnology (Table 1).

The newly emerging synthetic biology approaches may offer some clues for developing competitive technology for industrial biotechnology to produce “high volume and low price” products (Table 2). At the same time, bio-processing should try to become as similar as the chemical industry, including the need to develop continuous and open fermentation processes for e.g. making biofuels and PHA bioplastics [10-12]. Also, from now and toward a distant future, foods are still important for feeding the world population, the development of bioprocesses based on kitchen waste or activated sludge as substrates may also be an important option for a competitive industrial biotechnology (Table 2).

**Table 2:** Problems to be solved for making industrial biotechnology competitive to chemical technology

**Problems**	**Weakness of Industrial biotechnology**	**Possible solutions**
Microorganisms grow too slow	Slow: production takes days	Minimizing the microbial cells
Microbes can not use mixed substrates	Agricultural products are mostly mixed substrates	Assembling pathways that can metabolize mixed substrates
Low conversion of substrates to products	Cell metabolism turn substrates into CO_2_, H_2_O & byproducts	Removing unnecessary pathways consuming substrates
High Consumption on fresh H_2_O	Fresh H_2_O as medium et al.	Utilization of sea water for cell growth
Microbial cells grow to very low density	Product concentration low: Several mg to 100 g/L	Minimizing oxygen demand for aerobic cells & reducing Quorum sensing effects
Discontinuous processing	Contamination concerns	Developing continuous process
Sterilization costs high	High pressed steam	Contamination resisting strains grown in open systems
High energy demand for intensive aeration	Aerobic microorganisms need a lot of oxygen for growth	Developing anaerobic bioprocesses
Difficulty to control the bio-processes	Complicated cellular metabolisms	Artificial cells that contain only necessary metabolic pathways
One product by one microbial organism	Different organism has different strength.	Development of a platform organism for many products
Organisms consume food related products	Food for Fuels (Chemicals)	Kitchen wastes or activated sludge as substrates
Production facility costly	Costly materials and sensors	The use of carbon steel facilities et al.

Combination of bio- and chemical processes can offer a lot of advantages including bio-based (CO_2_ reduction) and fast reaction. Typical example includes the bio-production of lactic acid from anaerobic fermentation that is very effective and has only one single lactic acid product, and chemical polymerization of lactide to polylactide (PLA), a biodegradable green plastic [2,13]. The PLA story is a successful combination of bio- and chemical advantages. Others like succinic acid and 1,4-butanol bio-production and their copolymerization are under intensive R&D [2,13]. However, at the end, commercial successes have to be dependent on economy.

## Competing interests

The author declares that he has no competing interests.

## References

[B1] Chen GQ Kazlauskas R Chemical biotechnology in progress Curr Opin Biotechnol 2011 22 1 2 10.1016/j.copbio.2010.12.002 21862309

[B2] Chen GQ Patel M Plastics derived from biological sources: Present and future - A technical and an environmental review Chemical Rev 2012 112 2082 2099 10.1021/cr200162d 22188473

[B3] Chen GQ A Polyhydroxyalkanoates Based Bio- and Materials Industry Chem Soc Rev 2009 38 2434 2446 10.1039/b812677c 19623359

[B4] Gao X Chen JC Wu Q Chen GQ Polyhydroxyalkanoates as a source of chemicals, polymers and biofuels Curr Opin Biotechnol 2011 22 1 7 10.1016/j.copbio.2010.12.002 21705209

[B5] Hemple F Bozarth AS Lindenkamp N Klingl A Zauner S Linne U Steinbuchel A Maier UG Microalgae as bioreactor for bioplastic production Microb Cell Fact 2011 10 81 10.1186/1475-2859-10-81 22004563 PMC3214846

[B6] Tripathi L Wu LP Chen GQ Microbial synthesis of diblock copolymer poly-3-hydroxybutyrate-block-poly-3-Hydroxyhexanoate [P(3HB)-b-P(3HHx)] by a genome reduced *Pseudomonas putida* KT2442 Microb Cell Fact 2012 11 44 10.1186/1475-2859-11-44 22480145 PMC3442986

[B7] Zhou XY Yuan XX Shi ZY Meng DC Jiang WJ Wu LP Wu Q Chen JC Chen GQ Hyperproduction of poly(4-hydroxybutyrate) from glucose by recombinant *E. coli* Microb Cell Fact 2012 11 54 10.1186/1475-2859-11-54 22550959 PMC3527305

[B8] Zhang S Wang ZH Chen GQ Microbial polyhydroxyalkanote synthesis repression protein PhaR as an affinity tag for recombinant protein purification Microb Cell Fact 2010 9 28 10.1186/1475-2859-9-28 20459707 PMC2873406

[B9] Wang ZH Ma P Chen J Zhang J Yao YC Zhang HF Chen GQ A heterogeneous two-hybrid system in *Escherichia coli* based on polyhydroxyalkanoates synthesis regulatory proteins PhaR Microb Cell Fact 2011 10 21 10.1186/1475-2859-10-21 21477323 PMC3079617

[B10] Zhang XJ Luo RC Wang Z Deng Y Chen GQ Applications of (R)-3-hydroxyalkanoate Methyl Esters Derived from Microbial Polyhydroxyalkanoates as Novel Biofuel Biomacromolecules 2009 10 707 711 10.1021/bm801424e 19249855

[B11] Tan D Xue YS Gulsimay Chen GQ Unsterile and Continuous Production of Polyhydroxybutyrate by *Halomonas* TD01 Bioresource Technol 2011 102 8130 8136 10.1016/j.biortech.2011.05.068 21680179

[B12] Johnson K Jiang Y Kleerebezem R Muyzer G van Loosdrecht MCM Enrichment of a Mixed Bacterial Culture with a High Polyhydroxyalkanoate Storage Capacity Biomacromolecules 2009 10 670 676 10.1021/bm8013796 19193058

[B13] Lee JW Kim HU Choi S Yi J Lee SY Microbial production of building block chemicals and polymers Curr Opin Biotechnol 2011 22 758 767 10.1016/j.copbio.2011.02.011 21420291

